# Efficacy of pharmacological and non-pharmacological interventions for the treatment of anorexia nervosa in adolescents and adults (EfaNosa): protocol for a network meta-analysis

**DOI:** 10.1186/s13643-025-02999-6

**Published:** 2025-12-09

**Authors:** Franziska Halter, Almut Zeeck, Armin Hartmann, Christian Fleischhaker, Barbara Haack-Dees, Timo Brockmeyer, Ulrich Cuntz, Stefan Ehrlich, Alessio Maria Monteleone, Marco Solmi, Julia Stadelmaier, Eva Kiesswetter, Maria Petropoulou, Heidrun Janka, Maria-Inti Metzendorf, Joerg J. Meerpohl, Lukas Schwingshackl, Angela M. Kunzler

**Affiliations:** 1 Institute for Evidence in Medicine, Medical Center - University of Freiburg / Medical Faculty - University of Freiburg, Freiburg, Germany; 2 Cochrane Germany, Cochrane Germany Foundation, Freiburg, Germany; 3 Department for Psychosomatic Medicine and Psychotherapy, Medical Center-University of Freiburg, Medical Faculty - University of Freiburg, Freiburg, Germany; 4 Department of Child and Adolescent Psychiatry, Psychotherapy, and Psychosomatics, Medical Center-University of Freiburg, Medical Faculty - University of Freiburg, Freiburg, Germany; 5 Clinical Psychology and Translational Psychotherapy, Department of Psychologys, University of Münster, Münster, Germany; 6 Schön Klinik Roseneck, Prien, Germany; 7 Division of Psychological and Social Medicine and Developmental Neurosciences, Faculty of Medicine, Technische Universität Dresden, Dresden, Germany; 8 Eating Disorder Research and Treatment Center,Department of Child and Adolescent Psychiatry, Faculty of Medicine, Technische Universität Dresden, Dresden, Germany; 9 Department of Psychiatry, University of Campania L. Vanvitelli, Naples, Italy; 10 Department of Psychiatry, University of Ottawa, Ottawa, ON Canada; 11 Department of Mental Health, The Ottawa Hospital, Ottawa, ON Canada; 12 Ottawa Hospital Research Institute (OHRI), Clinical Epidemiology Program, University of Ottawa, Ottawa, ON Canada; 13 School of Epidemiology and Public Health, Faculty of Medicine, University of Ottawa, Ottawa, ON Canada; 14 Department of Child and Adolescent Psychiatry, Charité Universitätsmedizin, Berlin, Germany; 15 Institute of Medical Biometry and Statistics, Medical Center - University of Freiburg, Medical Faculty - University of Freiburg, Freiburg, Germany; 16 Institute of General Practice (ifam), Medical Faculty of the Heinrich - Heine - University Düsseldorf, Düsseldorf, Germany; 17 Clinical Psychology and Psychotherapy for Children and Adolescents, Institute for Psychology, Technische Universität Braunschweig, Braunschweig, Germany

**Keywords:** Anorexia nervosa, Eating disorders, Systematic review, Meta-analysis, Network meta-analysis, Treatment, Adult, Adolescent, Drug therapy, Psychotherapy

## Abstract

**Background:**

Anorexia nervosa (AN) is a severe eating disorder. With a lifetime prevalence of 1.4% in women and 0.2% in men, an increasing incidence and the highest mortality rate of all mental disorders, it is recognized as a major public health threat. Although the number of available treatments is increasing worldwide, the comparative efficacy and safety of different treatment options have not been investigated systematically. In this systematic review with network meta-analysis (NMA), we aim to assess the efficacy and safety of pharmacological and non-pharmacological interventions in adolescents and adults with AN.

**Methods:**

We will consider randomized controlled trials investigating pharmacological (e.g., antidepressants) and non-pharmacological (e.g., cognitive behavioral therapy) interventions compared to each other and to relevant control groups (e.g., no treatment, treatment as usual, waiting list) in adolescents (≥ 10 years) and/or adults (≥ 18 years) with AN. Eligible outcomes will include physiological (e.g., body weight) and psychological (e.g., depressive symptoms) outcomes prioritized by various interest-holders, including patients and healthcare professionals. Six electronic databases will be searched (Ovid MEDLINE, Scopus, WHO Global Index Medicus, Cochrane Central Register of Controlled Trials, Science Citation Index Expanded [Web of Science], Ovid APA PsycINFO). We will additionally search for grey literature and unpublished trials in further sources. Study selection, data extraction and the risk of bias assessment (Cochrane RoB 2 tool), will be performed independently by two reviewers. We will synthesize the data using a random-effects network meta-analysis and component network meta-analysis, if appropriate. We will assess inconsistency using a random-effects design-by-treatment interaction model. As effect measures, the risk ratio will be used for dichotomous outcomes, while the (standardized) mean difference will be utilized for continuous outcomes. Subgroup analyses, sensitivity analyses, and an assessment of publication bias are planned. The certainty of evidence derived from NMA will be assessed using the Grading of Recommendations, Assessment, Development and Evaluation (GRADE) approach.

**Discussion:**

Our NMA will provide important findings on the efficacy and safety of (non-)pharmacological interventions, offering valuable insights to inform clinical guidelines for the treatment of AN. The findings will inform various interest-holders including patients, their families, and clinical decision-makers.

**Systematic review registration:**

PROSPERO 2025 CRD420250654515.

**Supplementary Information:**

The online version contains supplementary material available at 10.1186/s13643-025-02999-6.

## Background

Anorexia nervosa (AN) is a severe eating disorder that is associated with considerable physical, mental, and social impairments. With a lifetime prevalence of 1.4% for women, 0.2% for men, and a point prevalence of 5.7% in adolescents [1], AN is widely recognized as a major public health threat. According to the Global Burden of Disease (GBD) study, the prevalence increases around the age of 15 years, especially in females [2]. During the first wave of the COVID-19 pandemic, a higher number of new diagnoses and hospitalizations in children and adolescents was observed [3]. In addition, a landmark meta-analysis [4] found a standardized mortality ratio for AN of 5.9 deaths per 1000 person-years, an almost sixfold increased risk, highlighting the need for effective treatments.

Several evidence-based guidelines have been developed to inform the treatment of different eating disorders, including AN [e.g., 5–11]. For children and adolescents, these guidelines consistently recommend the involvement of near caregivers, mostly in the form of family-based treatment or therapy (FBT) [5]. For adults, the main treatment recommendation is individual psychotherapy, with no superiority of one approach over another [5, 12, 13]. All guidelines highlight the lack of evidence concerning pharmacotherapy for AN, especially as the sole treatment [5]. Previous international guidelines were developed using literature searches from 2013 to 2022, with the World Federation of Societies of Biological Psychiatry (WFSBP) guideline [11] currently being the most recent one.

To the best of our knowledge, there are five relevant published systematic reviews with and without network meta-analysis (NMA) [11, 12, 14–16] and one umbrella review [13] evaluating the efficacy of different treatments for AN in adolescents and adults in inpatient and outpatient settings (see Additional file 1). These reviews [e.g., [15] informed clinical guidelines on the treatment of AN [e.g., 6], but have some methodological limitations. First, except for Himmerich et al. [11] and Zhu et al. [16], who searched until January or July 2022, literature searches of previous reviews were mostly conducted until the end of 2020. Therefore, these reviews did not consider several new randomized controlled trials (RCTs) with large samples and/or long-term follow-up on (non-)pharmacological interventions for adolescents and adults that have been published in recent years [e.g., 17–19]. In addition, RCTs on newer treatment options like neurostimulation, which are still in its infancy [e.g., 20–27], were also not considered. Second, there is no information on the comparative efficacy of pharmacological and non-pharmacological interventions, partly due to a different focus of the reviews [11, 15, 16], inaccurate reporting of eligible studies in the reviews [13, 14], or insufficient data to investigate those types of interventions in an NMA [12]. Third, previous reviews have mostly focused on weight outcomes, while a meta-review of 13 qualitative evidence syntheses found that patients prioritize psychological needs over physical recovery [28], highlighting the need to investigate additional outcomes including mental health (e.g., depressive symptoms, health-related quality of life [HRQoL]). Lastly, an exploration of subgroup differences between patients (e.g., female and male patients, patients with and without comorbidities) is urgently needed as subgroup analyses were rarely performed. Overall, patients, their families and various interest holders like healthcare staff would benefit from an updated systematic review to inform the revision of guidelines regarding the treatment of AN.

Therefore, this systematic review with NMA aims to examine the comparative efficacy and safety of various pharmacological and non-pharmacological interventions in the treatment of AN in adolescents and adults, and to obtain a clinically meaningful ranking of different treatment alternatives compared to each other and to relevant control groups, such as no treatment, treatment as usual and waiting list. To prevent problems encountered by other reviews [12, 13], we will perform a component network meta-analysis (CNMA), allowing us to estimate the effects of individual components of complex (i.e., multicomponent) interventions.

## Methods

This protocol for a systematic review with network meta-analysis is reported according to the Preferred Reporting Items for Systematic reviews and Meta-Analyses for Protocols (PRISMA-P) [29]. The PRISMA-P checklist can be found in Additional file 2. The NMA will be reported according to the Preferred Reporting Items of Systematic reviews and Meta-Analyses for Network Meta-Analyses (PRISMA-NMA) [30]. The protocol has been registered in the International Prospective Register of Systematic Reviews (PROSPERO) database on 24th February 2025 (CRD420250654515).

### Eligibility criteria

The eligibility criteria are defined according to the Population, Intervention, Comparator, Outcome, Study design (PICOS) framework. A detailed description of the eligibility criteria is provided in Table 1.

**Table 1:** Eligibility criteria

Component	Inclusion		Exclusion
Population	• Adolescents (≥ 10 years) and/or adults (≥ 18 years) diagnosed with AN, using internationally recognized diagnostic criteria (i.e., DSM-IV TR/DSM-5; ICD-10/ICD-11): - Significantly low body weight for individual’s height, age, developmental stage, or weight history (i.e., adolescents: BMI-for-age < 5th percentile; adults: BMI < 18.5 kg/m^2^)^a^ - Adults: Rapid weight loss (> 20% of total body weight within 6 months) instead of low body weight as essential feature if other criteria are met - Adolescents: Failure to gain weight as expected based on individual developmental trajectory • Mixed populations (i.e., AN and other eating disorders) if outcomes for AN are analyzed and reported separately		• Children (< 10 years) • Adolescents/adults with other mental disorder than AN • Mixed populations if outcomes for AN are not analyzed and reported separately
Intervention^b^	• Pharmacological and non-pharmacological treatments delivered in any setting (i.e., inpatient, day hospital, outpatient) and independent of the duration of the intervention - Pharmacological treatments: (1) Antidepressants (i.e., tricyclic antidepressants, selective Serotonin Reuptake Inhibitors [SSRIs]) (2) Antipsychotics (i.e., olanzapine, risperidone) (3) Other pharmaceuticals (i.e., appetite stimulants, lithium, D-cycloserine, anxiolytics – benzodiazepines, oxytocin, growth hormone, psychedelic drugs) - Non-pharmacological treatments: (1) Psychotherapy, including: (a) Approaches in line with German directives for psychotherapy: Cognitive behavioral therapy (CBT) including CBT-E, Psychodynamic therapy including Focal psychodynamic psychotherapy (FPT) (b) Further (evidence-based) psychotherapeutic approaches: Family-based treatment (FBT), Systemic family therapy (SyFT), Specialist Supportive Clinical Management (SSCM), Maudsley Model of Anorexia Nervosa Treatment for Adults (MANTRA) (c) Other psychotherapeutic approaches: e.g., Interpersonal therapy (IPT), Dialectic-behavioral therapy (DBT), Mentalization-based treatment (MBT), Client-centered psychotherapy, Integrative approaches, Body-oriented methods, combination of distinct psychotherapeutic approaches (d) Other treatment approaches and methods: e.g., Cognitive remediation therapy (CRT), Cognitive Bias Modification (CBM), Exposure-based approaches Various modalities of psychotherapy (e.g., face-to-face/digital; guided/self-help; individual/group) (2) Neuromodulation (e.g., deep brain stimulation) (3) Physical activity-related and exercise-based interventions (4) Dietary supplements (e.g., omega-3 fatty acids) (5) Novel refeeding approaches • Complex (i.e., multicomponent) interventions combining any of the above treatments (e.g., psychotherapy plus olanzapine)		None
Comparator	• Treatment as usual (TAU), waiting list, no intervention (i.e., inactive control), different pharmacological and non-pharmacological treatments outlined above		None
Outcomes^c,d,e^	Prioritized outcomes for which we will perform the certainty of evidence assessment using GRADE: 1. Body weight (kg)/BMI (kg/m^2^); for adolescents: age- and sex-specific BMI percentile 2. Global eating disorder psychopathology (i.e., Eating Disorder Examination/questionnaire [EDE/-Q] total score including restraint, eating concern, shape concern, and weight concern) 3. Body image disturbance (e.g., Body Image Questionnaire) 4. All-cause mortality (i.e., total number of deaths from any cause during the trial period, that is, intervention and follow-up duration) 5. Depressive symptoms (e.g., Beck Depression Inventory) 6. HRQoL (e.g., Short Form-12, or eating disorder specific, e.g., Engel quality of life instrument) 7. Adverse events (e.g., headache, electrolyte abnormalities, metabolic disorders, suicidal ideation, as defined by trialists)	Additional outcomes: 8. Acceptance of therapy/dropouts (i.e., dropout rates per group during treatment) 9. Daily weight gain (kg) 10. Recovery/remission (e.g., ≥ 95% ideal body weight, by DSM-5 or trialist-defined cut-off on standardized scale measure for remission vs. no remission) 11. Self-esteem (e.g., Rosenberg Self-esteem Scale) 12. Neuropsychological outcomes (e.g., cognitive flexibility using Wisconsin Card Sorting Test) 13. Social functioning (e.g., Work and Social Adjustment Scale) 14. Days in hospital (only inpatient or day hospital setting)	None
Study design	RCTs: • including at least two study arms (i.e., intervention vs. control or vs. intervention) • reporting on one or more of the above outcomes • with a minimum follow-up period of six months or more • with parallel-group or crossover design (for crossover trials, we will only consider the pre-crossover endpoints for the analyses)		• Non-randomized trials • RCTs without a follow-up period of at least six months

*AN* anorexia nervosa, *BMI* Body Mass Index, *CBT* cognitive behavioral therapy, *CBT-E* enhanced CBT, *CRT* cognitive remediation therapy, *DBT* dialectic-behavioral therapy, *DSM-IV TR/DSM-5* Diagnostic and Statistical Manual of Mental Disorders 4^th^ Edition Text Revision [57]/5^th^ Edition [58], *FBT* family-based treatment/therapy, *FPT* focal psychodynamic psychotherapy, *GRADE* Grading of Recommendations, Assessment, Development and Evaluation, *HRQoL* health-related quality of life, *ICD-10/11* International Statistical Classification of Diseases and Related Health Problems 10th Revision [59]/11th Revision [60], *IPT* interpersonal therapy, *MANTRA* Maudsley Model of Anorexia Nervosa Treatment for Adults, *MBT* mentalization-based treatment, *NMA* network meta-analysis, *RCT* randomized controlled trial, *SSCM* Specialist Supportive Clinical Management, *SSRIs* selective serotonin reuptake inhibitors, *SyFT* systemic family therapy, *TAU* treatment as usual

^a^For the ICD-11, due to the prognostic relevance, a differentiation between two types according to the amount of underweight is suggested: AN with significantly low body weight (BMI > 14.0 kg/m^2^ and < 18.5 kg/m^2^ or between 0.3^rd^ and 5^th^ age percentile) and with dangerously low body weight (BMI < 14.0 kg/m^2^ or < 0.3^rd^ percentile), with a distinction between a restrictive type and a “binge-purging type” in each group

^b^Further relevant interventions may be added in the review development process

^c^The first seven outcomes, for which we will assess the certainty of evidence and provide a summary of findings table (see “Synthesis of included studies” section), were provisionally prioritized by clinical experts involved in this review. This prioritization might be adjusted based on the planned patient workshops (see “Eligibility criteria” section). Moreover, we will examine additional outcomes specified here

^d^To ensure comparability of the results, the outcome measurements will be evaluated at harmonized time points (e.g., 12 months), and the last available time point of follow-up

^e^The first three outcomes have been mapped to the criteria on AN in DSM-5

#### Population

We will include studies that assessed adolescents (≥ 10 years) and/or adults (≥ 18 years) diagnosed with AN. Mixed populations (i.e., AN and other eating disorders) will be considered if outcomes for the defined populations are analyzed and reported separately.

#### Intervention

Studies evaluating pharmacological (e.g., antidepressants) and/or non-pharmacological treatments (e.g., psychotherapy) in any setting and of any intervention duration will be considered. As non-pharmacological interventions, we will also include trials evaluating neuromodulation, physical activity-related and exercise therapy interventions, nutritional supplements, and novel refeeding approaches. Complex (i.e., multicomponent) interventions will also be considered.

#### Comparator

In our NMA, interventions will be compared, where possible, to each other and to relevant control groups, such as no treatment, treatment as usual and waiting list.

#### Outcomes

 We will include studies reporting outcomes that have been suggested by clinical experts involved in the current review (AZ, AH, CF, BHD, TB, UC, SE, AMM, MS) or that have been considered in previous systematic reviews and clinical guidelines. In addition, we plan to identify further potentially patient-relevant outcomes by involving patients with AN in the review development process using a peer-research format. Ideally, two workshops with adolescent and adult participants (*n* = 8 each), currently in treatment for AN or former patients, will be held at the Medical Center, University of Freiburg. In these workshops, we will explain the importance of our evidence synthesis project to the participants. Subsequently, we plan to use discussions to obtain further information about the patients’ experiences. Patients will discuss relevant outcomes of interventions, which will be summarized. Overall, based on these sources, we will provide a Summary of findings table for the first seven prioritized outcomes based on the assessment of the certainty of evidence (see “Synthesis of included studies” section). The first seven outcomes specified in Table 1 were provisionally prioritized by the clinical experts in this review (AZ, AH, CF, BHD, TB, UC, SE, AMM, MS), comprising weight-related and psychological outcomes mapping the DSM-5 criteria for AN, as well as possible adverse events (for details, see Table 1). This prioritization will be adjusted, if necessary, based on the results of the patient workshops.

#### Study design

We will include RCTs using a parallel-group or crossover design (see “Synthesis of included studies”) to examine interventions of any duration. RCTs have to include a minimum follow-up period of 6 months or more after the intervention. If more than one article is published on the same study, we will identify the original publication and will extract multiple outcomes from different papers, including the largest number of participants and using data from intention-to-treat analysis (ITT; see “Synthesis of included studies” section). We will use no restrictions concerning publication date or language. We will consider published RCTs and grey literature (e.g., unpublished theses).

### Information sources and literature search

The following six databases will be searched: Ovid MEDLINE, Scopus, World Health Organization (WHO) Global Index Medicus, Cochrane Central Register of Controlled Trials (CENTRAL), Science Citation Index Expanded (Web of Science), and Ovid APA PsycInfo. We will not include Embase and Cumulative Index to Nursing and Allied Health Literature (CINAHL) in our search, as RCTs indexed here are now prospectively added to CENTRAL via a highly sensitive screening process. As additional sources, we will screen the reference lists of systematic reviews and of included studies to identify further eligible trials. To retrieve grey literature, we will search the Bielefeld Academic Search Engine (BASE). Moreover, we will search two clinical trial registries (ClinicalTrials.gov, WHO International Clinical Trials Registry Platform [ICTRP]) to identify planned, ongoing or completed but unpublished studies. The strategy was developed by an experienced information specialist (HJ) and quality checked by a second experienced information specialist (MIM). It includes three clusters of search terms: a) terms related to AN, b) different treatments, and c) study design. We will use validated search filters for RCTs, if possible [e.g., 31]. The search strategy for Ovid MEDLINE is provided in Additional file 3 and will be translated and adapted to all sources.

### Study selection process

Identified references will be saved in EndNote. We will use Cochrane’s Screen4Me workflow to help assess the search results [32]. After excluding duplicates, the references will be uploaded to Covidence (www.covidence.org) for title–abstract and full-text screening. Two reviewers (i.e., FH, JS, EK, LS, and AMK in different dual combinations) will independently screen the titles and abstracts of the studies. If an abstract is not provided by the database from which it originated and the title appears potentially relevant, we will forward the record to the full-text review stage. At this stage, the full-text versions of potentially relevant studies will be retrieved or acquired, and will also be screened by two reviewers independently. At both levels of screening, the two reviewers will compare their lists of relevant studies that meet the inclusion criteria. Potential disagreements will be discussed. If no consensus can be found or reached, they will seek the opinion of a third author (AZ, JJM, or LS). We will present a PRISMA flow diagram to show the process of study selection [33]. We will list all articles excluded after full-text assessment and will provide the reasons for exclusion [33]. For identified planned or ongoing trials, we will provide a table to follow up on these trials.

### Data extraction and data collection process

Two reviewers (i.e., FH, JS, EK, and AMK in different dual combinations) will independently extract the data for each included study using a pre-tested excel data extraction form. We will extract information on design/methods, participants, interventions/comparators, and outcomes (see Additional file 4). Any disagreements will be discussed and if no agreement can be reached, we will consult a third reviewer (AZ, JJM, or LS) to find a consensus. If relevant data are missing, we will contact the corresponding study authors to request missing information (e.g., to ask for post-intervention data if only change from baseline is reported).

### Risk of bias assessment

The risk of bias (RoB) will be assessed by two independent reviewers (i.e., FH, JS, EK, and AMK in different dual combinations) for each outcome using the revised Cochrane risk-of-bias tool for randomized trials (RoB 2) [34]. This tool enables a domain-level judgment about the risk of bias comprising the following domains: bias arising from the randomization, bias due to deviations from intended interventions, bias due to missing outcome data, bias in measurement of the outcome, and bias in selection of the reported results. For this review, we are mainly interested in the effects of assignment to intervention (i.e., intention-to-treat effects). Any disagreements in the risk of bias assessment will be resolved by discussion or by consulting a third reviewer (JJM or LS). The RoB for each domain will be judged as ‘low risk’, ‘high risk’, or ‘some concerns’ by applying the code of the RoB 2 tool and by using the available RoB 2 Excel sheet [35]. Based on these results, we will establish an overall risk-of-bias judgment for each predefined outcome. A traffic light plot of the domain-level judgments for each individual study and weighted bar plots of the distribution of risk-of-bias judgments within each domain will be provided using the R package “robvis” [36].

### Synthesis of included studies

**Fig. 1:**
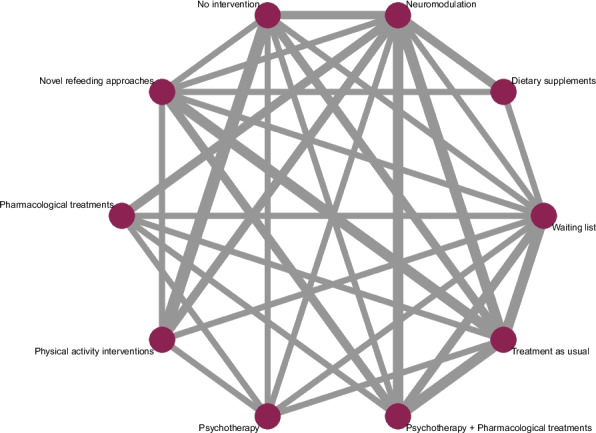
Example network plot

The statistical analysis has been planned and will be conducted by an experienced statistician (MP). We will conduct an NMA with the aim of assessing several comparative interventions (e.g., psychotherapy, pharmacological treatments, TAU) by combining direct and indirect evidence from RCTs. A frequentist random-effects NMA will be performed in order to evaluate the summary effects of each intervention using the R package “netmeta” [37, 38]. As effect measures, we will use the risk ratio (RR) for dichotomous outcomes (e.g., remission), the mean difference (MD) for continuous outcomes (e.g., BMI), or the standardized mean difference (SMD) for continuous outcomes reported on different scales (e.g., HRQoL, BMI). For crossover trials, we will only consider the pre-crossover endpoints for the main analyses to avoid carry-over effects, especially in trials on pharmacological interventions. Data from ITT will be preferred over completer or per-protocol analyses. We will not consider any post-hoc analyses. We will provide a network plot (Fig. 1) [2]; an example network plot for hypothetical data) to visually depict the direct comparisons between the different interventions and control groups for each outcome [39].

To summarize NMA results, effect estimates with their 95% confidence intervals (CIs) will be presented in league tables and forest plots. Treatments will be ranked by P-scores that are a frequentist version of the Surface Under the Cumulative Ranking curve (SUCRA) [40]. *P*-scores are values between 0 and 1, with 1 indicating that a treatment always ranks best and a value of 0 meaning that a treatment always ranks worst. If at least ten comparisons are available for a particular outcome, we will evaluate the presence of small-study effects for each outcome using comparison-adjusted funnel plots. Comparison-adjusted funnel plots will also allow detecting extreme study effects (i.e., outliers) [41], which can be further explored with outlier analysis using the R package “NMAoutlier” [42].

The fundamental assumption of NMA is transitivity, meaning that the distribution of potential effect modifiers is balanced across treatment comparisons. We will assess this empirically by comparing the distribution of a-priori defined potential effect modifiers across the direct comparisons. The statistical manifestation of transitivity is consistency, which requires that direct and indirect evidence is in agreement. We will assess inconsistency as a whole in each network using a random-effects design-by-treatment interaction model [43, 44]. If the global test suggests inconsistency, we will assess inconsistency locally by splitting the direct and the indirect evidence using the loop-specific approach [45, 46] and, visually, using the net-heat plot [47]. A-priori defined potential effect modifiers are based on previous reviews [e.g., 15] and include the following variables:

1. the patients’ age (i.e., young adolescents [10–14 years] vs. adolescents [15–19 years] vs. young adults [20–24 years] vs. 25–29 years vs. 30–34 years vs. 35–39 years vs. ≥ 40 years) [48, 49];
2. sex;
3. the duration of disease (i.e., number of years since diagnosis);
4. subtype of AN (e.g., restricting vs. binge-eating/purging type based on DSM-5 and ICD-11);
5. chronicity of disease (i.e., below vs. over 7-year cut-off for severe and enduring AN) [50];
6. severity of disease (i.e., weight status based on ICD-11: AN with significantly low body weight [BMI 14.0–18.5 kg/m^2^ in adults; 0.3-5^th^ percentile for BMI-for-age in adolescents] vs. AN with dangerously low body weight [BMI < 14.0 kg/m^2^ in adults; < 0.3 percentile for BMI-for-age in adolescents]; adapted to ethnicity, if possible);
7. previous treatment (i.e., any previous eating disorder-specific pharmacological or non-pharmacological treatment vs. not);
8. comorbidity of mental illness (i.e., with comorbid mental disorder vs. without);
9. treatment setting (e.g., inpatient vs. day hospital vs. outpatient);
10. modality of psychotherapeutic interventions (e.g., face-to-face vs. digital interventions, group vs. individual);
11. duration of the intervention (in weeks);
12. study design (i.e., parallel-group studies vs. crossover trials, where only pre-crossover data will be considered for crossover trials);
13. duration of follow-up after the intervention (i.e., ≤ 6 months vs. > 6 months ≤ 1 year vs. > 1 year ≤ 3 years vs. > 3 years ≤ 5 years vs. > 5 years);
14. risk of bias of included studies;
15. the provision of ITT analyses by included studies; and
16. the type of reported data (i.e., post-intervention vs. change-from-baseline data).

If appropriate, these potential effect modifiers will be incorporated into the analyses by using meta-regression and subgroup analyses (i.e., for age, sex, subtype of AN, chronicity/severity of disease, previous treatment, comorbidity, treatment setting, modality of psychotherapeutic interventions, study design, duration of follow-up) to explore their impact on treatment effects. In addition, we will perform sensitivity analyses by excluding studies with different key characteristics that contribute to heterogeneity (i.e., only including studies rated as low RoB, only including studies providing ITT analyses, only including studies reporting post-intervention data).

The complexity of the interventions is a major challenge, as this can lead to increased heterogeneity if not accounted for properly. As we expect to end up with a network including complex (i.e., multicomponent) interventions in AN, for example, of CBT and olanzapine, we plan to conduct a CNMA to estimate the effect of individual components of multicomponent interventions [51]. We will perform the additive CNMA model if the additivity assumption is satisfied. Additionally, we will explore various interaction CNMA models to account for potential interactions (e.g., more efficient use of CBT interventions due to olanzapine-induced reduced motor unrest) among components [51, 52].

We will follow the GRADE (Grading of Recommendations, Assessment, Development and Evaluation) approach for NMA to assess the certainty of evidence in each of the direct, indirect and network estimates [53]. Two review authors (i.e., FH, JS, EK, and AMK in different dual combinations) will independently rate the certainty of evidence for each of the seven outcomes prioritized above (see “Eligibility criteria” section and Table 1). We will resolve any differences in assessment by discussion or by consultation with a third review author (JJM or LS). To evaluate direct evidence, we will consider the risk of bias, inconsistency, indirectness, and publication bias. If the certainty of direct evidence is high and its contribution (i.e., relative weight in the network estimate) to the network estimate is at least as much or more than that of the indirect evidence, we will not rate the indirect evidence [53]. To assess the certainty of network estimates, we will compare the ratings of direct and indirect evidence. If we detect inconsistency in a specific pairwise comparison, we will rate down the network estimate and acknowledge its limitations. If NMA or pairwise meta‐analyses are not possible, we will present the results in a narrative format in the Summary of findings table.

## Discussion

Given the ambivalence of patients with AN to engage in treatment [54], the willingness of patients and, in the case of adolescents, also of their relatives to consider therapy may substantially depend on the advice provided by specialized staff (e.g., clinicians). Therefore, high-quality evidence on the various treatment alternatives and their comparative efficacy and safety is essential.

This systematic review with NMA aims to examine the efficacy and safety of different pharmacological and non-pharmacological interventions delivered in various settings and modalities, compared to each other, where possible, and to control groups. Supplementing previous systematic reviews, it will use extended eligibility criteria and consider new relevant RCTs in the field, involving patients and important interest holders to identify patient-relevant outcomes and to disseminate the results using appropriate forms to reach patients, their families, clinical decision-makers and further interest holders (e.g., self-help groups).

The findings of this systematic review with NMA will inform the revision of the German clinical guideline [6], which is currently being worked on and possibly further international guidelines that need to be revised. The NMA will allow comparing interventions that might have never been compared before as well as to rank interventions based on their efficacy and safety. Therefore, our results have the potential to play a crucial role in improving clinical care in inpatient, day-hospital and outpatient treatment settings and, thus, the quality of life of adolescent and adult patients with AN and their families.

Based on previous research [e.g., 15, 55, 56] and the importance of multidisciplinary treatment approaches highlighted by several clinical guidelines [5], it can be expected that the included studies, especially those in inpatient care, often investigate complex (i.e., multicomponent) interventions in patients with AN. Such cases include a treatment consisting of several (possibly interacting) components like psychotherapy, pharmacotherapy (e.g., single or several medications), and further treatment elements (e.g., nutritional interventions). This makes it challenging to distinctly separate individual components, such as splitting treatment nodes to conduct separate systematic reviews on the efficacy of pharmacological and non-pharmacological interventions. Furthermore, especially for emerging treatments (e.g., neuromodulation), it remains unclear if these treatments should be delivered as stand-alone or adjunctive interventions [e.g., 27]. Therefore, we decided to examine both pharmacological and non-pharmacological interventions in one review using an NMA and CNMA approach to analyze their effects and to assess not only which interventions do work, but also which components. Providing a ranking at both intervention and component levels may optimize the use of resources in clinical care and, thus, improve patient outcomes in the treatment of AN.

## Supplementary Information

Additional file 1. Characteristics of previous research. This file presents the characteristics of relevant previous research, including systematic reviews and one umbrella review, on the treatment of AN in adolescents and adults. These reviews were considered in planning and designing the current review.

Additional file 2. PRISMA-P checklist. This supplement includes the Preferred Reporting Items for Systematic reviews and Meta-Analyses for Protocols (PRISMA-P) checklist for reporting this protocol.

Additional file 3. Search strategy for Ovid MEDLINE. This file contains the search strategy for Ovid MEDLINE as an example for the search strategy of this review.

Additional file 4. Items for data extraction. This supplement presents the items for the data extraction sheet that will be used to extract relevant data from the included studies.

## Data Availability

Not applicable since protocol for a review. All predefined forms to generate and analyze data (e.g., data extraction sheet to extract data from included studies) are included in this protocol and its supplementary information files.

## Abbreviations

AN: Anorexia nervosa
APA: American Psychiatric Association
BASE: Bielefeld Academic Search Engine
BMI: Body Mass Index
CBT(-E): (enhanced) Cognitive behavioral therapy
CENTRAL: Cochrane Central Register of Controlled Trials
CI: Confidence interval
CINAHL: Cumulative Index to Nursing and Allied Health Literature
CNMA: Component network meta-analysis
COI: Conflict of interest
CRT: Cognitive remediation therapy
DBT: Dialectic-behavioral therapy
DMS-IV TR/DSM-5: Diagnostic and Statistical Manual of Mental Disorders, 4^th^ Edition Text Revision / 5^th^ Edition
FBT: Family-based treatment / therapy
FPT: Focal psychodynamic psychotherapy
GBD: Global Burden of Disease
GRADE: Grading of Recommendations, Assessment, Development and Evaluation
HRQoL: Health-related quality of life
ICD-10/-11: International Statistical Classification of Diseases and Related Health Problems 10th Revision/11th Revision
ICTRP: WHO International Clinical Trials Registry Platform
IPT: Interpersonal therapy
IQR: Interquartile range
ITT: Intention-to-treat analysis
MANTRA: Maudsley Model of Anorexia Nervosa Treatment for Adults
MBT: Mentalization-based treatment
MD: Mean difference
MeSH: Medical Subject Heading
NMA: Network meta-analysis
No.: Number
PICOS: Population, intervention, outcome, comparator, study design
PRISMA-NMA: Preferred Reporting Items of Systematic reviews and Meta-Analyses for Network Meta-Analyses
PRISMA-P: Preferred Reporting Items for Systematic reviews and Meta-Analyses for Protocols
PROSPERO: International Prospective Register of Systematic Reviews
RCT: Randomized controlled trial
RoB: Risk of Bias
RR: Risk ratio
SD: Standard deviation
SE: Standard error
SMD: Standardized mean difference
SSCM: Specialist Supportive Clinical Management
SSRIs: Selective Serotonin Reuptake Inhibitors
SUCRA: Surface Under the Cumulative Ranking curve
SyFT: Systemic family therapy
TAU: Treatment as usual
WFSBP: World Federation of Societies of Biological Psychiatry
WHO: World Health Organization
