# Supplementary material for: Efficacy of pharmacological and non-pharmacological interventions for the treatment of anorexia nervosa in adolescents and adults (EfaNosa): protocol for a network meta-analysis
Source: Syst Rev. 2025 Dec 9;14:245. doi: 10.1186/s13643-025-02999-6 (PMC12687480; doi:10.1186/s13643-025-02999-6)
Supplement: Supplementary file 1 — Additional file 1. Characteristics of previous research. This file presents the characteristics of relevant previous research, including systematic reviews and one umbrella review, on the treatment of AN in adolescents and adults. These reviews were considered in planning and designing the current review. [file 13643_2025_2999_MOESM1_ESM.docx]

**Additional file 1. Characteristics of previous research**

| Author and year | Review type | Search date | PICOS | | | | | | | Main results |
| --- | --- | --- | --- | --- | --- | --- | --- | --- | --- | --- |
|  |  |  | Population | | Intervention | | Comparator | Outcomes | Studies |  |
|  |  |  | Diagnosis | Age group | Types of intervention | Treatment setting |  |  |  |  |
| Zeeck et al., 2018 | SR with NMA | 11/2005 –02/2017 | AN | Adolescents | PT | Inpatient; day hospital/  day clinic; outpatient | FBI^a^ | Body weight/BMI | 10 RCTs:  (2^nd^ measurement within 3 years after intervention) | FBI were slightly more effective than other psychotherapeutic approaches |
|  |  |  |  | Adults |  |  | SSCM^a^ |  | 11 RCTs^b^:  (2^nd^ measurement within 3 years after intervention) | Several psychotherapeutic interventions can be recommended; no superiority of one psychotherapeutic approach over another |
| Murray et al., 2019 | SR with MA | 01/1980 – 12/2017 | AN (participants without core symptoms were excluded, e.g., non-fat-phobic anorexia ) | Adolescents | Specialized interventions (PHARMA and NON-PHARMA) | Inpatient;  outpatient;  mixed setting | Placebo; active intervention; non-specific intervention;  TAU | Weight outcomes;  psychological symptoms | 9 RCTs assessing at least 2 time points | *Results across all population groups:* specialized interventions were more effective than comparator interventions concerning weight outcomes at the end of intervention, but not at follow-up;  no effects were found for psychological  symptoms |
|  |  |  |  | Adults |  |  |  |  | 8 RCTs assessing at least 2 time points |  |
|  |  |  |  | Mixed population |  |  |  |  | 18 RCTs assessing at least 2 time points |  |
| Solmi et al., 2021 | SR with NMA | until 03/2020 | Acute AN (no relapse prevention trials) | Adults | NON-PHARMA;  PHARMA | Outpatient | TAU | Weight;  BMI;  global eating disorder psychopathology;  dropouts | 13 RCTs assessing at least 2 time points | Specific psychological interventions compared to TAU are associated with modest improvements regarding clinical course and quality of life;  no superiority of one psychotherapeutic approach over another |
| Monteleone et al., 2022 | Umbrella review | until 12/2020 | Any eating disorder | Adolescents | PT (individual, group, self-help)  PHARMA;  psycho-education;  physical exercise | Inpatient;  outpatient;  mixed | Active  (PHARMA and NON-PHARMA); TAU; placebo; WL; NT; mixed TAU and WL/NT;  mixed  active and WL/NT | ED-specific behaviors;  neuropsychological functioning;  ED-specific psychopathology;  functioning and quality of life; general psychiatric  symptoms;  global course of the disease;  weight/BMI | 4 MAs & NMAs | FBI in outpatient setting was more effective than active control regarding remission |
|  |  |  |  | Adults |  |  |  |  | 7 MAs & NMAs | FBI was more effective than active interventions for remission and weight gain |
|  |  |  |  | Mixed population |  |  |  |  | 9 MAs & NMAs | FBI improved the global course of the disease compared to TAU and to active interventions;  hormones improved weight/BMI compared to placebo |
| Himmerich et al. 2023 | SR with guideline update | 01/2011 – 01/2022 | Any eating disorder | Children; adolescents; adults | PHARMA | Inpatient;  day care program;  outpatient;  mixed;  research center | Placebo; no control | Weight-related outcomes; ED symptoms, mood;  other psychiatric disorders (e.g., anxiety), social functioning; physical symptoms | 70 articles (RCTs, open studies, phase 2 or 3 studies, case series, case reports) & 4 MAs | Strong evidence for the effectiveness of olanzapine with a limited recommendation for its use; limited or low evidence for other drugs, some with a recommendation against their use |
|  |  |  |  |  |  |  |  |  |  |  |
| Zhu et al. 2023 | SR with MA (Cochrane review) | until 07/2022 | Severe and enduring anorexia nervosa (duration of > 3 years) | Children (< 16 years) | Specific PT (e.g., SSCM; FBI) | Inpatient; day-patient; outpatient | PT (specific or non-specific); NT; WL; TAU; PHARMA; dietary counselling | Clinical improvement (e.g., weight restoration); treatment non-completion; clinical response (e.g., ED symptoms); HRQoL; participant satisfaction; dropouts | 2 RCTs | It is unclear if there is a difference between CBT-SEAN compared to SSCM-SE on clinical improvement at 12 months or treatment non-completion |
|  |  |  |  | Adults and older adolescents (≥ 16 years) |  |  |  |  |  |  |

*Note.* AN: anorexia nervosa; BMI: Body Mass Index; CBT-SEAN: cognitive behavioral therapy for severe and enduring anorexia nervosa; ED: eating disorder; FBI: Family-based intervention; HRQoL: Health‐related quality of life; MA: meta-analysis; NMA: network meta-analysis; NON-PHARMA: non-pharmacological intervention; NT: no treatment; PHARMA: pharmacological intervention; PT: Psychotherapeutic interventions; SR: systematic review; SSCM: Specialist Supportive Clinical Management; SSCM-SE: Specialist Supportive Clinical Management for severe and enduring anorexia nervosa; TAU: treatment as usual; WL: waiting list.

^a^ For NMAs, each intervention is compared to each other; the interventions presented in this column were defined as ‘central comparator’ by the review authors.

^b^ Three of these studies were excluded for NMA.
