# Supplementary material for: Efficacy of pharmacological and non-pharmacological interventions for the treatment of anorexia nervosa in adolescents and adults (EfaNosa): protocol for a network meta-analysis
Source: Syst Rev. 2025 Dec 9;14:245. doi: 10.1186/s13643-025-02999-6 (PMC12687480; doi:10.1186/s13643-025-02999-6)
Supplement: Supplementary file 3 — Additional file 3. Search strategy for Ovid MEDLINE. This file contains the search strategy for Ovid MEDLINE as an example for the search strategy of this review. [file 13643_2025_2999_MOESM3_ESM.docx]

**Additional file 3. Search strategy for Ovid MEDLINE**

**Ovid MEDLINE(R) ALL**

Database: Ovid MEDLINE(R) ALL <1946 to February 04, 2025>

1. Anorexia Nervosa/
2. Anorexia/
3. (anorexia or anorexias or anorectic$).ti,ab,kf.
4. or/1-3
5. (((pharmaco* or medical) adj3 (therap* or treat* or intervent*)) or pharmacotherap*).ti,ab,kf.
6. (antidepressant$ or antipsychotic$ or mood stabili*).ti,ab,kf.
7. (psychological therap$ or psychotherap$).ti,ab,kf.
8. ((psychodynamic* or cognitive or dialectial or behavio?ral) adj3 therap$).ti,ab,kf.
9. (family adj3 (treatment$ or therap* or intervent*)).ti,ab,kf.
10. (acipimox or alendronate or arginine or valprazolam or chlorophenylpiperazine or cyprohep-tadine or cbd or dehydroepiandrosterone or dronabinol or paroxetine or sertraline or fluoxe-tine or escitalopram or cisapride or citalopram or fluvoxamine or venlafaxine or desvenlafaxine or mirtazapine or naltrexone or duloxetine or D-cycloserine or trazodone or vortioxetine or agomelatine or dapoxetine or clomipramine or lithium carbonate or nortriptyline or amitriptyline or imipramine or desipramine or doxepin or atomoxetine or aripiprazole or asenapine or quetiapine or risperidone or teriparatide or chlorpromazine or zuclopenthixol or clozapine or olanzapine or risedronate or tyrosine).ti,ab,kf.
11. hormon*.ti,kf. or (hormon* adj3 (treatment$ or therap* or intervent*)).ti,ab,kf.
12. (estrogen or ghrelin or progestin or testosterone or oxytocin or adrenal or gonadal).ti,ab,kf.
13. (refeed* or re-feed* or sham feed*).ti,ab,kf.
14. ((nutrition* adj1 intervent*) or (nutrition adj2 therap*) or (diet adj2 therap*) or (diet* adj1 supplement*)).ti,ab,kf.
15. (neurostimulation$ or brain stimulation$ or transcranial magnetic stimulation$).ti,ab,kf.
16. ((digital or online or internet or web or website$ or web-site$ or computer* or smartphone) adj3 (intervention$ or therap$ or support* or care or after-care or self-help or training or application* or app?)).ti,ab,kf.
17. or/5-16
18. randomized controlled trial.pt.
19. controlled clinical trial.pt.
20. randomized.ab.
21. placebo.ab.
22. drug therapy.fs.
23. randomly.ab.
24. trial.ab.
25. groups.ab.
26. or/18-25
27. exp animals/ not humans.sh.
28. 26 not 27
29. Anorexia/dh [Diet Therapy]
30. Anorexia/dt [Drug Therapy]
31. Anorexia/th [Therapy]
32. Anorexia Nervosa/dh [Diet Therapy]
33. Anorexia Nervosa/dt
34. Anorexia Nervosa/th
35. or/29-34
36. 28 and 35
37. 4 and 17 and 28
38. 36 or 37
