# Supplementary material for: Efficacy of pharmacological and non-pharmacological interventions for the treatment of anorexia nervosa in adolescents and adults (EfaNosa): protocol for a network meta-analysis
Source: Syst Rev. 2025 Dec 9;14:245. doi: 10.1186/s13643-025-02999-6 (PMC12687480; doi:10.1186/s13643-025-02999-6)
Supplement: Supplementary file 4 — Additional file 4. Items for data extraction. This supplement presents the items for the data extraction sheet that will be used to extract relevant data from the included studies. [file 13643_2025_2999_MOESM4_ESM.docx]

**Additional file 4. Items for data extraction**

| Category | Data items | |
| --- | --- | --- |
| Study information & PICOS summary | - First author, author list and corresponding author - Registries - Full citation including title, year, source - Language - Publication type (e.g., journal, dissertation) - Publication/registration date - Study aim/purpose - Study setting (e.g., inpatient, day hospital, outpatient) - PICOS summary | |
| Study details | - Country - World bank classification - Recruitment period - Sources of recruitment - Study duration - Duration of follow up (based on post-test/the end of intervention) - Single or multi-center - Parallel-group or crossover trial - No. of study arms - No. of randomized clusters (if available) - No. of randomized participants (*N*) - No. of participants receiving intervention (*n*) - No. of dropouts and no. of analyzed participants (*n*) - Definition of analysis population (e.g., per protocol, ITT) - Funding - Conflict of interest (COI) | |
| Population characteristics | - Inclusion/exclusion criteria - Subgroups (relevant for review) reported (yes/no) - Baseline age (mean, SD, median, range, IQR) - Sex (i.e., proportion of female participants) - Ethnicity - Socioeconomic status - Pre-existing (comorbid) conditions beyond AN (proportion and specified; e.g., mental illness) - Previous treatment (e.g., pharmacological or non-pharmacological) - Mean baseline BMI - Severity of disease (based on weight status/BMI) - Definition of AN (e.g., subtype like restricting vs. binge-eating/purging type) - Duration of AN (i.e., number of years since diagnosis), including chronicity (e.g., 7-year cut-off for severe and enduring AN) | |
| Interventions | *Pharmacotherapy:*   - Name - Dose - Frequency and duration (e.g., in weeks) - Route of administration - Treatment setting (e.g., inpatient, day hospital, outpatient) - Compliance with intervention (e.g., study flow diagram) | *Non-pharmacological interventions:*   - Form/category of intervention (e.g., psychotherapy, neurostimulation, physical activities) - Name - Frequency - Duration (e.g., in weeks) - Treatment setting (e.g., inpatient, day hospital, outpatient) - Intervention modality (e.g., group, individual; face-to-face, online, mixed) - Delivered by - Content of intervention - Compliance with intervention (e.g., study flow diagram) |
|  | - Co-intervention - Treatment crossovers (e.g., study flow diagram) | |
| Comparators | *Pharmacotherapy:*   - Name (e.g., placebo) - Usual/standard care - Compliance with control (e.g., study flow diagram) | *Non-pharmacological interventions:*   - Form/category of comparator (e.g., no treatment, waitlist) - Name - Frequency - Duration (e.g., in weeks) - Comparator setting (e.g., inpatient, day hospital, outpatient) - Comparator modality (e.g., group, individual; face-to-face, online, mixed) - Delivered by - Content of comparator group - Compliance with control (e.g., study flow diagram) |
| Outcome summary | - Primary or secondary outcome (as defined by study) - Outcome definition/name (as defined by study) - Outcome measurement (i.e., assessment instrument used) - Measurement time point - Outcome assessor - Type of available outcome data (i.e., post-intervention, change-from-baseline data) - Type of outcome (e.g., binary, continuous) - For continuous: interpretation of outcome direction - Analysis population - All or subgroup - Maximum duration of follow-up - Source(s) for outcome data | |
| Outcome data (binary/ categorical) | - Intervention/control arm, respectively: no. of events - Intervention/control arm, respectively: no. of participants analyzed - Intervention/control arm, respectively: observed events - Intervention/control arm, respectively: no. of patients analyzed - Risk ratio (RR) with 95% CI | |
| Outcome data (continuous) | - Intervention/control arm, respectively: mean at baseline - Intervention/control arm, respectively: SD for mean at baseline (or SE, 95% CI) - Intervention/control arm, respectively: no. of participants analyzed at baseline - Intervention/control arm, respectively: mean at post-test - Intervention/control arm, respectively: SD for mean at post-test (or SE, 95% CI) - Intervention/control arm, respectively: no. of participants analyzed at post-test - Intervention/control arm, respectively: mean difference (MD) from baseline - Intervention/control arm, respectively: SD for mean difference (or SE, 95% CI) - Between-group mean difference (post score) - SD for between-group mean difference (post score) (or SE, 95% CI) - Between-group mean difference (change score) - SD for between-group mean difference (change score) (or SE, 95% CI) | |

*Note.* AN: anorexia nervosa; BMI: body mass index; CI: confidence interval; COI: conflict of interest; IQR: interquartile range; ITT: intention-to-treat analysis; no.: number; PICOS: population, intervention, comparator, outcome, study design; RR: risk ratio; SD: standard deviation; SE: standard error.
